# Melittin Restores PTEN Expression by Down-Regulating HDAC2 in Human Hepatocelluar Carcinoma HepG2 Cells

**DOI:** 10.1371/journal.pone.0095520

**Published:** 2014-05-02

**Authors:** Hui Zhang, Bin Zhao, Cheng Huang, Xiao-Ming Meng, Er-Bao Bian, Jun Li

**Affiliations:** 1 School of pharmacy, Anhui key laboratory of bioactivity of natural products, Anhui Medical University, Hefei, Anhui Province, China; 2 Institute for Liver Diseases of Anhui Medical University (AMU), Hefei, Anhui Province, China; Institut für Pathologie, Greifswald, Germany, Germany

## Abstract

Melittin is a water-soluble toxic peptide derived from the venom of the bee. Although many studies show the anti-tumor activity of melittin in human cancer including glioma cells, the underlying mechanisms remain elusive. Here the effect of melittin on human hepatocelluar carcinoma HepG2 cell proliferation *in vitro* and further mechanisms was investigated. We found melittin could inhibit cell proliferation *in vitro* using Flow cytometry and MTT method. Besides, we discovered that melittin significantly downregulated the expressions of CyclinD1 and CDK4. Results of western Blot and Real-time PCR analysis indicated that melittin was capable to upregulate the expression of PTEN and attenuate histone deacetylase 2 (HDAC2) expression. Further studies demonstrated that knockdown of HDAC2 completely mimicked the effects of melittin on PTEN gene expression. Conversely, it was that the potential utility of melittin on PTEN expression was reversed in cells treated with a recombinant pEGFP-C2-HDAC2 plasmid. In addition, treatment with melittin caused a downregulation of Akt phosphorylation, while overexpression of HDAC2 promoted Akt phosphorylation. These findings suggested that the inhibitory of cell growth by melittin might be led by HDAC2-mediated PTEN upregulation, Akt inactivation, and inhibition of the PI3K/Akt signaling pathways.

## Introduction

Hepatocellular carcinoma (HCC) is the fifth most common cancer in the world without effective therapies [1], [2]. Although the most effective treatment of HCC is surgery, recurrence-free 5-year survival after curative resection is low [3]. So new remedies would possibly depend on advances in basic research.

Phosphatase and tensin homolog (PTEN) is a plasma membrane lipid phosphatase that acts as a tumor suppressor and it dephosphorylates PIP3 to PIP2, inhibits the activation of the oncogene Akt and then negatively regulates the PI3K/Akt pathway [4], [5]. PI3K/Akt signaling is one of the best characterized pathways targeted by PTEN through its lipid phosphatase activity and important in regulating growth, survival and proliferation of cells [6]. Emerging evidence has shown that HDAC inhibitors up-regulate PTEN expression by promoting histone acetylation at its promoter in lung cancers and brain cancers [7], [8].

The histone deacetylases (HDACs) represent an ancient superfamily of enzymes conserved from yeast to human. The HDAC members of class I contains HDAC1, HDAC2, HDAC3 and HDAC8 [9]. Histone acetylation and deacetylation of nucleosomal core histones play an important roles in modulation of chromatin structure and regulation of gene expression. Disruption of balance between histone acetyltransferases (HATs) and histone deacetylases (HDACs) is known to be involved in the carcinogenesis [10]. Different types of HDAC overexpression have been detected in various human cancers, like human colorectal cancer, stomach and liver cancer [11]–[13]. In fact, the inhibition of HDACs using various known HDAC inhibitors exhibited antitumor activities in HCC model systems including HCC-derived cell lines or murine models [14], [15].

Melittin is a water-soluble toxic peptide and the major component of bee venom [16]. There have been several advances in the development of its anti-bacterial, anti-viral, anti-inflammatory and anti-cancer effective [16], [17]. Now, the progression of cancer evolvement is no longer thought to be emerged only by genetic alterations, but also should be acknowledged as a result of epigenetic alterations. Histone deacetylation represents the most important epigenetic modification responsible for chromatin remodeling. Consequently, histone deacetylases inhibitors are becoming the new class of potent anti-cancer drugs.

In light of the therapeutic potential of melittin in HepG2 cells, our study was performed to elucidate the biological mechanism by which melittin induces the inhibition of cell growth in HepG2 cells. Here, we hypothesized that melittin leads to inhibition of cell proliferation and up-regulation of PTEN gene which may be associated with the decrease of HDAC2 expression in HepG2 cell.

## Materials and Methods

### Materials

Melittin was purchased from Baichun Anhui co., Ltd, batch number: 20061216 (Anhui, china). Antibodies against CyclinD1, CDK4 were purchased from Boster (Wuhan, China) or β-actin was purchased from Santa Cruz Biotechnology (California, USA), Akt antibodies, phospho-Akt antibodies, H3 antibodies and ac-H3 antibodies were purchased from Cell Signaling (Beverly, MA, USA). HDAC2 antibody was purchased from anbo (San Francisco, CA, USA). HDAC2, cyclinD1, CDK4 and PTEN primers were produced from Shanghai Sangon Biological and Technological Company (Shanghai, China). Secondary antibodies for goat anti-rabbit immunoglobulin IgG horse radish peroxidase (HRP), and goat anti-mouse IgG HRP were purchased from Santa Cruz Biotechnology (Santa Cruz, California, USA).

### Cell culture and treatment

Hepatocellular carcinoma cell (HepG2) was obtained from Biopharmaceutical research institute, Anhui Medical University. HepG2 cells were cultured in Dulbecco's modified Eagle's medium (DMEM, Gibco, USA), supplemented with 100 U/ml penicillin, 100 mg/ml streptomycin, 2 mML-glutamine, and 10% fetal calf serum. Cell cultures were maintained at 37°C at an atmosphere of 5% CO_2_. Melittin was dissolved in dimethylsulfoxide (DMSO) and treated to cells. The final DMSO concentration was not exceeded 0.1% (v/v).

### Cell proliferation assay

Cellular proliferation was measured using MTT assay. 5×10^3^cells were seeded in 96-well plates at 37°C in a humid chamber with 5% CO_2_. The cells were treated with TSA and 1, 4 and 8 µg/ml of melittin in media containing 1% FBS for 0, 12, 24 and 48 h. 5 mg/ml MTT was then added to each well and incubated with cells at 37°C for 4 h. the medium was replaced and the formazan crystals were dissolved in 150 µl dimethylsuophoxide (DMSO). The optical density (OD) was determined with Thermomax microplate reader (Bio-TekEL, USA) at 490 nm wavelenth. All experiments were performed in triplicate and repeated at least three times.

### Cell cycle assay

HepG2 cells were seeded in 6-well plate. Cells were trypsinized and collected in ice-cold PBS. Cells were resuspended in 300 µL PBS and fixed by adding 700 µL of frozen 100% ethanol, then incubated overnight at −20°C. After fixation, cells were washed with cold PBS and stained with 0.5 ml of propidium iodide (PI) staining buffer, which contained 200 mg/ml RNase A, 50 µg/ml PI, at 37°C for 30 min in the dark. Analyses were performed on BDLSR flowcytometer (BD Biosciences). The experiments were repeated three times.

### RT-PCR

Total RNA was extracted from human HepG2 cell using TRIzol reagents (Invitrogen). The first strand cDNA was synthesized from total RNA using Thermoscript RT-PCR System (Takara) according to the manufacturer's instructions. RT-PCR was carried out understanding protocol using the primers (Table 1). PCR was performed at 94°C for 5 min, following by 30 or 35 cycles of amplification at 94°C for 40 s, 51 or 60°C for 40 s and 72°C for 1 min by using ABI9700. The band intensities were measured by a densitometer and the results were normalized with β-actin. All experiments were performed in triplicate and repeated at least three times.

**Table 1:** Primer sequences used for reverse transcriptase polymerase chain reaction.

Target RNA	Accession No.	Primer	Sequence
HDAC2	NM001527.3	forward	5′-ATAAAGCCACTGCCGAAGAA-3′
		reverse	5′-TCCTCCAGCCCAATTAACAG-3′
PTEN	NM000314.4	forward	5′-GCTAGCCTCTGGATTTGACG-3′
		reverse	5′-ACCAGGACCAGAGGAAACCT-3′
CyclinD1	NM053056.2	forward	5′-CTCCTGGTGAACAAGCTCAAGT-3′
		reverse	5′-GCGGTAGTAGGACAGGAAGTTG-3′
CDK4	NM000075.3	forward	5′-GGGACCGTCAAGCTGGCTGA-3′
		reverse	5′-TCGAGGCCAGTCGTCTTCTG-3′
β-actin	NM001101.3	forward	5′-CCCACACTGTGCCCATCTACG-3′
		reverse	5′-GCCATCTCTTGCTCGAAGTCC-3′

### Quantitative real-time PCR

Relative levels of specific mRNA were determined with a SYBR Green using quantitative real-time PCR detection system (Themal 5100) according to the manufacturer's instruction. PCR was performed at follows: 95°C for 5 min, then 40 cycles of 95°C for 15 s, 60°C for 30 s, 72°C for 30 s, and then a final extension at 72°C for 30 s. Relative expression levels were calculated according to the standard 2−ΔΔCT method using β-actin gene as endogenous control for normalization. Quantitative real-time PCR was carried out with standard protocol using the primers (Table 1). All experiments were performed in triplicate and repeated at least three times.

### Western blot

The whole cell extracts were prepared, and protein concentration of samples was determined by using a BCA protein assay kit (Boster, China). Whole-cell extracts were then fractionated by electrophoresis through a 12% sodium dodecyl sulf-polyacrylamide gel electrophoresis (SDS-PAGE). Gels were run at a 120 V for 2 h before being transferred on to a PVDF membrane (Millipore Corp., Billerica, MA, USA). After blockade of nonspecific protein binding, nitrocellulose blots were incubated for 1 h with primary antibodies diluted in TBS/Tween20 (0.075%) containing 3% Marvel. Anti-phospho-Akt, anti-Akt, H3 and ac-H3 were diluted 1∶1000. Human monoclonal antibodies directed against PTEN, HDAC2 or β-actin were used at 1∶300 and 1∶500. Following incubation with primary antibodies, blots were washed four times in TBS/Tween-20 before incubation for 1 h in goat anti-mouse or anti-rabbit horse radish peroxidase conjugate antibody at 1∶10000 dilution in TBS/Tween-20 containing 5% skim milk. After extensive washing in TBS/Tween-20, the blots were processed with distilled water for detection of antigen using the enhanced chemilumin escence system. Proteins were visualized with the ECL-chemiluminescent kit (ECL-plus, Thermo Scientific).

### RNA interference (RNAi) analysis

RNAi experiments in HepG2 cells were performed by forward transfection in 24 h cultured HepG2 using Lipofectamine RNAiMax (Invitrogen) according to the manufacturer's protocol. Small interfering RNA (siRNA) oligonucleotides against HDAC2 genes or scrambled sequences were synthesized by the Shanghai GenePharma Corporation. Transfection was allowed to proceed for various times and cells were processed for different assays. The siRNA transfection efficiency of Lipofectamine RNAiMax in cells was determined by the BlocK-iT Alexa Fluor Red Fluorescent Oligo protocol (Invitrogen). All experiments were performed in triplicate and repeated at least three times. The siRNA sequences were shown in Table 2.

**Table 2:** The sequences of siRNA Oligo.

	Sense	Antisense
HDAC2siRNA	5′-CCCAUAACUUGCUGUUAasdfAATT-3′	5′-UUUAACAGCAAGUUAUGGGTT-3′
Negative control	5′-UUCUCCGAACGUGUCACGUTT-3′	5′-ACGUGACACGUUCGGAGAATT-3′

### Generation of recombinant plasmid

Target gene HDAC2 was detected by reverse transcriptase polymerase chain reaction (RT-PCR). The amplified PCR products were electrophoresed on a 1% agarose gel and were visualized under UV light. The gel-purified PCR segment was cut with both Xho I and BamH I restriction enzymes, and then the segment was introduced into the pEGFP-C2 vector (stored in our laboratory) to form the pEGFP-C2-HDAC2 plasmid. The pEGFP-C2-HDAC2 plasmid was transformed into Tag 1 cells, and the correct fragment was identified by digestion with both Xho I and BamH I and was verified as the correct clone through sequence analysis. The constructed plasmid was transfected into HepG2 cells and observed under fluorescence microscopy assays. All experiments were performed in triplicate and repeated at least three times.

### Statistical analysis

Data are represented as mean±SE. Statistical analysis was performed by using ANOVA followed by Student's test. For changes in mRNA or protein levels, ratios of mRNA (relative expression) and protein (densitometric values) to respective house-keeping controls were compared. Significance was defined as p<0.05 or p<0.01.

## Results

### Inhibitory effect of melittin on the growth of HepG2 cell

**Figure 1:**
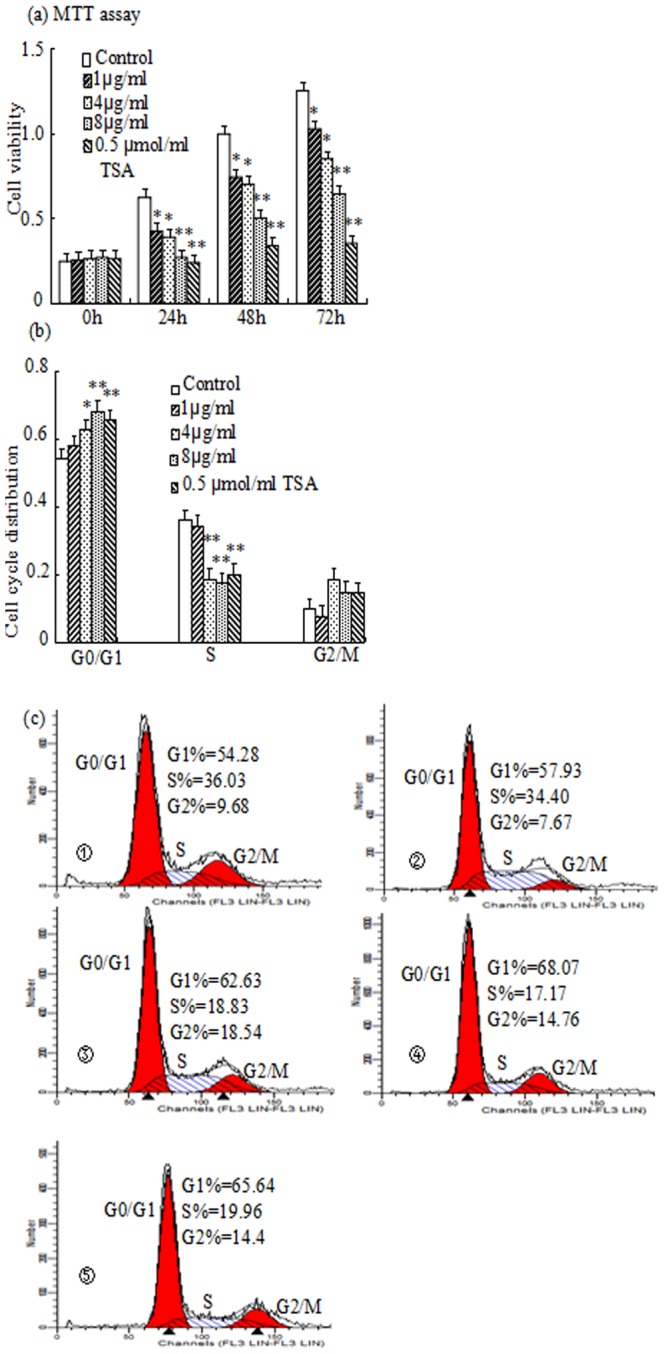
The effect of melittin on cell viability and cell cycle distribution in HepG2 cells were treated with 1, 4, 8 µg/ml of melittin and 0.5 µmol/ml TSA. Cell viability was assessed for 0, 24, 48 and 72 h by MTT (a), and melittin reduced cell viability in HepG2 cells in a dose-dependent manner. Results (mean ± SE) were calculated as percent of corresponding control values. Representative images of three independent experiments are shown. *P<0.05, **P<0.01, are significant. 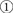 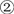 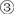 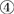 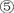 were Control, 1 µg/ml, 4 µg/ml, 8 µg/ml and 0.5 µmol/ml TSA, respectively. The percentages of cell cycle distribution was then evaluated by flow cytometric analysis (b). Cell cycle distribution was analyzed using the software supplied in the instrument (c). Representative blots and images of three independent experiments are shown. *P<0.05, **P<0.01 vs Control. Statistical analysis was performed by ANOVA.

To investigate whether melittin affected proliferation of HepG2 cells, HepG2 cells were treated with melittin and TSA for 0, 24, 48 and 72 h and the cell viability was measured. As shown in Fig 1a, HepG2 cells treated with 1, 4 and 8 µg/ml of melittin and 0.5 µmol/ml TSA reduced the cell growth by 31.7%, 37.0%, 57.8%, 59.7% in 24 h, 25.3%, 29.7%, 49.8%, 65.7% in 48 h and 18.1%, 32.3%, 48.6%, 71.8% in 72 h, respectively. The results indicated that melittin suppressed cell growth of HepG2 cells in dose-dependent manner. Because of the prominent inhibition of cell growth was observed at 24 h after melittin, so further study was performed by incubating the cells with melittin for 24 h.

### Melittin induces cell cycle arrest in HepG2 cell

Next, to study the potential mechanisms by which melittin suppressed HepG2 cells growth, the effect of melittin on the cell cycle progression was evaluated by flow cytometry. HepG2 cells were also treated with 1, 4 and 8 µg/ml of melittin as well as TSA for 24 h, and cell cycle distribution was then analyzed. As shown in Fig 1 b and c, melittin significantly enhanced the percentage of HepG2 cells in the G0/G1 phase in comparison to the control, at the same time, accompanied by a significant reduction of cell numbers in the S phase. Therefore, this result indicated that melittin affected the cell proliferation through interferencing G1 to S phase progression in cell cycle.

**Figure 2:**
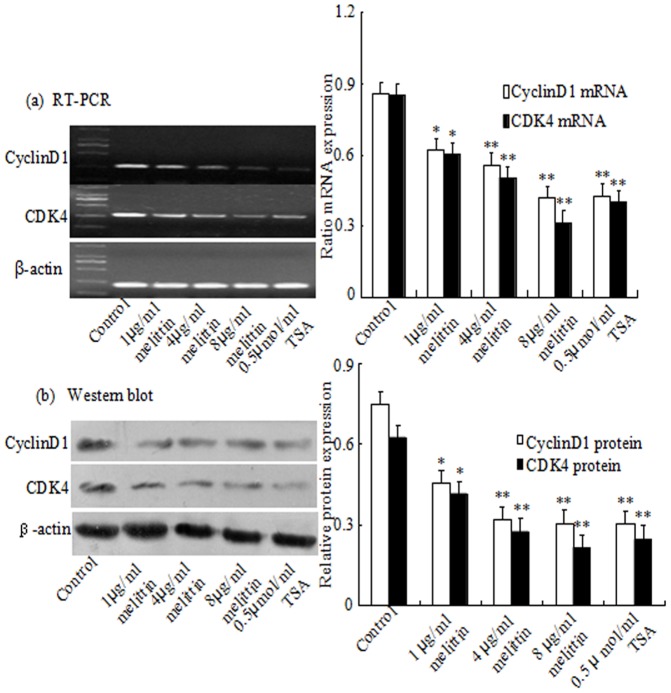
Effect of melittin on the expression of CyclinD1 and CDK4 in HepG2 cells. (a and b)Total cellular proteins and RNA were prepared and the expressions of CyclinD1 and CDK4 proteins and mRNA were analyzed using Western blot and RT-PCR. β-actin was used as an internal control. Representative blots and images of three independent experiments are shown. *P<0.05, **P<0.01 vs Control. Statistical analysis was performed by ANOVA.

In order to investigate the mechanisms by which melittin regulated the G0/G1 cell cycle arrest, the effects of melittin on the expression of G0/G1 cell cycle regulatory moleculars were examined by Western blot and RT-PCR analysis. CyclinD1 and CDK4 (Fig 2 a and b), which were participated in the G1 cell cycle progression, were markedly down-regulated under treatment, suggesting that melittin inhibited cell proliferation through G1 cell cycle arrest.

### Melittin restores tumor suppressor gene PTEN expression in HepG2 cell

**Figure 3:**
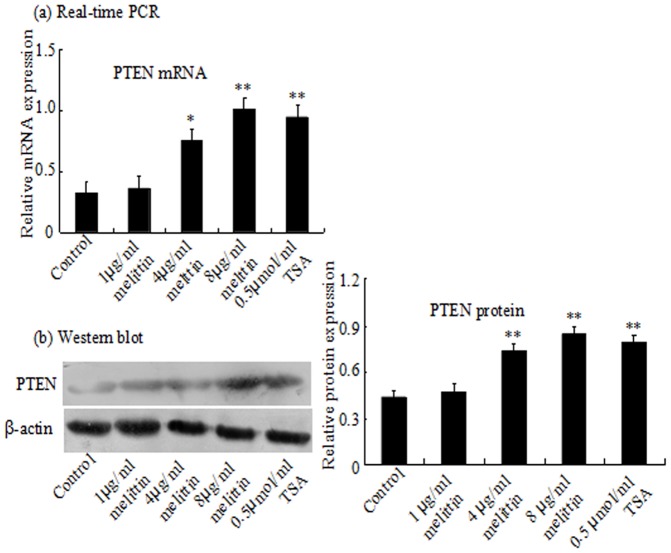
Effect of melittin on the expression of PTEN in HepG2 cells. The PTEN mRNA were analyzed by real-time PCR (a), and proteins expression was analyzed by Western blot(b). Melittin restores PTEN activity. All gels were representative of at least three independent experiments. *P<0.05, **P<0.01 vs Control.

PTEN is well known to regulate cell proliferation and survival. To validate whether the melittin-mediated cell proliferation is associated with PTEN expression, PTEN expression was examined by Real-time PCR and Western blot. As shown in Fig 3, we found the low expression of PTEN without treatment of melittin. Meanwhile, melittin increased the expression of PTEN in the range from 1 to 8 µg/ml in comparison to control in HepG2 cells. These results suggested that the inhibitory of cell proliferation by melittin may be associated with the upregulation of PTEN expression.

### Melittin downregulates histone deacetylase 2 expression in HepG2 cell

**Figure 4:**
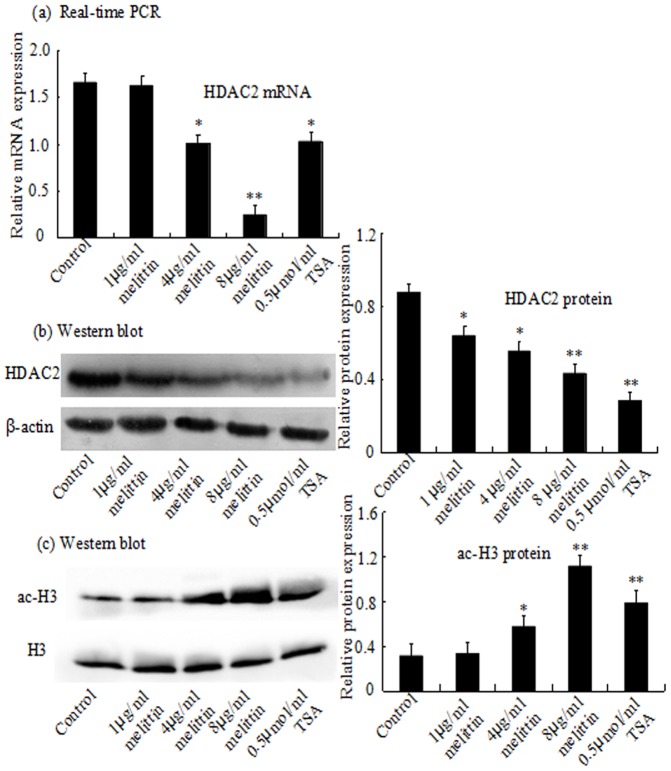
The inhibitory effect of melittin on expression of HDAC2 and in HepG2 cells. HepG2 cells were treated with 1, 4, 8 µg/ml of melittin and 0.5 µmol/ml TSA for 24 h. The HDAC2 mRNA expression were analyzed by real-time PCR (a). The HDAC2 and ac-H3 proteins expression were analyzed by Western blot (b) and (c). Representative images of at least three independent experiments are shown. *P<0.05, **P<0.01 vs Control.

**Figure 5:**
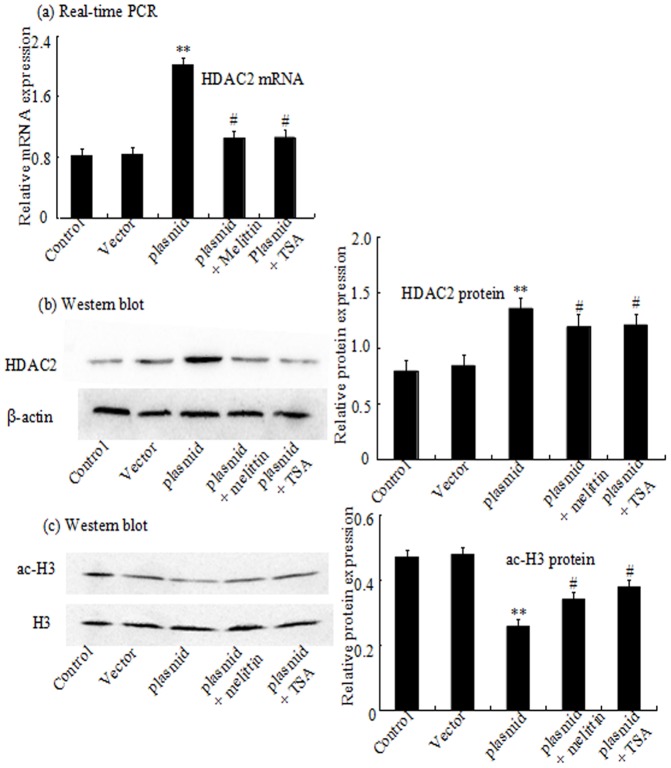
Effect of pEGFP-C2-HDAC2 plasmid on HDAC2 and histone H3 expression in HepG2 cells. HepG2 cells were treated with pEGFP-C2, pEGFP-C2-HDAC2 plasmid, pEGFP-C2-HDAC2 plasmid and melittin, pEGFP-C2-HDAC2 plasmid and TSA for 24 h. The mRNA expression of HDAC2 was analyzed by real-time PCR (a). The HDAC2 and ac-H3 proteins expression were analyzed by Western blot (b) and (c). Relative HDAC2 mRNA and protein levels are presented as mean ± standard of the mean (S.E.) of optical densities from three separated experiments. **P<0.01 vs control and empty pEGFP-C2 group. ^#^P<0.05 vs pEGFP-C2-HDAC2 plasmid group.

HDAC inhibitors have been reported to trigger cell cycle arrest. To investigate whether administration of melittin induces cell cycle arrest and inhibits cell proliferation in HDAC-dependant mechanisms, HepG2 cells was treated with melittin in the range from 1 to 8 µg/ml as well as TSA. As shown in Fig 4 a and b, HDAC2 was highly expressed in HepG2, but melittin reduced the expression of HDAC2 compared with the control group. Therefore, to further confirm for the inhibition of HDAC by melittin, intracellular change like the acetylation status of the histone protein H3 was detected. Western blot analysis indicated an increase of histone complex H3 acetylation after treatment (Fig 4 c). Moreover, compared with the high-level expression of endogenous HDAC2 in both the control group and the pEGFP-C2 vector group, HDAC2 expression in cells transfected with pEGFP-C2-HDAC2 plasmid was significantly enhanced. but this upregulation of HDAC2 by transfection of overexpression plasmid could be also largely blocked by the treatment of melittin or TSA (Fig 5 a and b), indicating that melittin may serve as a the potential novel HDAC inhibitor. In addition, it was demonstrated that the expression of the histone complex H3 acetylation was elevated companying with the downregulation of HDAC2. (Fig 5 c)

### Expression of PTEN is regulated by HADC2 in HepG2 cell

**Figure 6:**
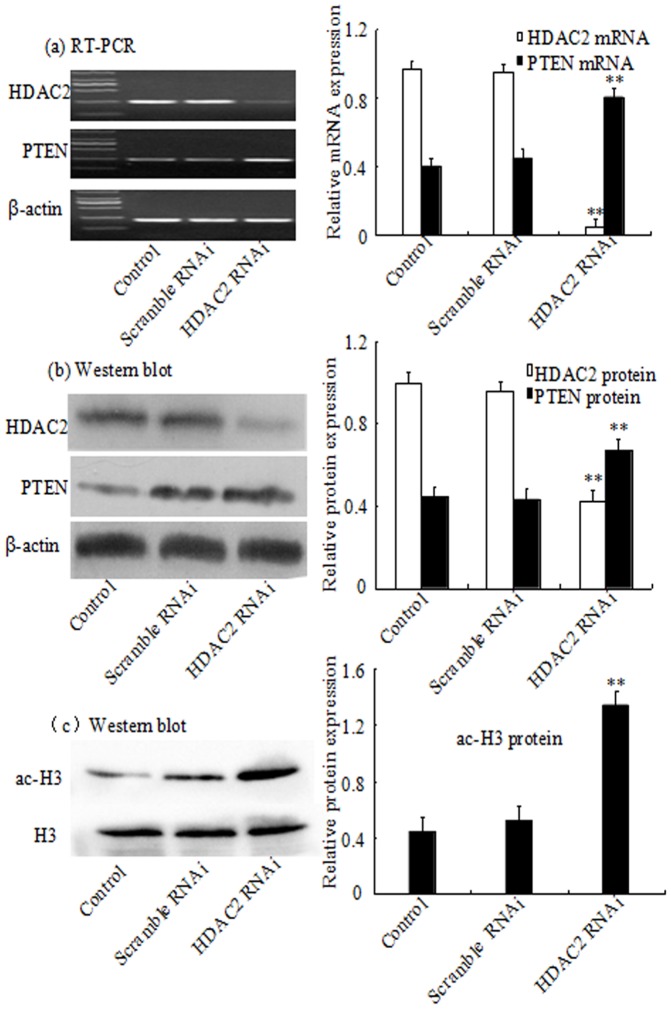
HDAC2 knockdown mimicked the effect of melittin in HepG2 cells. HepG2 cells were transfected with HDAC2 siRNA for 24 h. The HDAC2 and PTEN mRNA expressions were analyzed by RT-PCR (a). The HDAC2, PTEN and ac-H3 proteins expression was analyzed by Western blot (b) and (c). Representative images from 3 to 4 independent experiments are shown. **P<0.01 vs control and scrambled siRNA.

**Figure 7:**
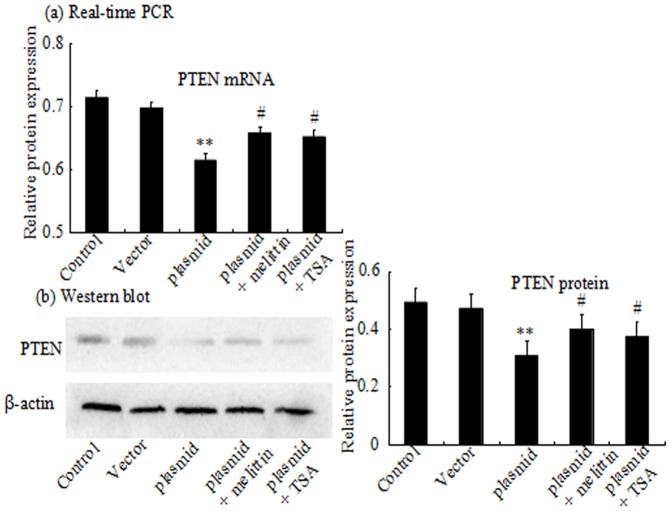
Effect of pEGFP-C2-HDAC2 plasmid on PTEN in HepG2 cells. HepG2 cells were treated with pEGFP-C2, pEGFP-C2-HDAC2 plasmid, pEGFP-C2-HDAC2 plasmid and melittin, pEGFP-C2-HDAC2 plasmid and TSA for 24 h. The PTEN mRNA expression was analyzed by real-time PCR (a). The PTEN protein expression was analyzed by Western blot (b). Relative PTEN mRNA and protein levels are presented as mean ± standard of the mean (S.E.) of optical densities from three separated experiments. **P<0.01 vs control and vector. ^#^P<0.05 vs pEGFP-C2-HDAC2 plasmid group.

To examine the function of PTEN in the HepG2 cells. The results of PTEN expression was intensified under TSA. At the same time, it was demonstrated that (Fig 6 a and b), the upregulation of PTEN expression was more significant in HDAC2 knockdown group compared with cells transfected with the scrambled siRNA. The expression of ac-H3 was substantially increased when HDAC2 was blocked (Fig 6 c). While PTEN expression was obviously decreased in HepG2 cells transfected with the pEGFP-C2-HDAC2 plasmid in comparison to the low-level expression of endogenous PTEN in HepG2 cells of both the control group and the pEGFP-C2 vector group(Fig 7 a and b). Compared to the plasmid group, PTEN expression was reversed under melittin or TSA, while the expression was still lower than the control group and the pEGFP-C2 vector group. These results suggested that there might be a deacetylation occurred in the PTEN gene and the inhibition of cell proliferation under melittin was associated with HDAC2.

### Melittin promotes HDAC-mediated PTEN activation through downregulation of Akt pathway on cell proliferation

**Figure 8:**
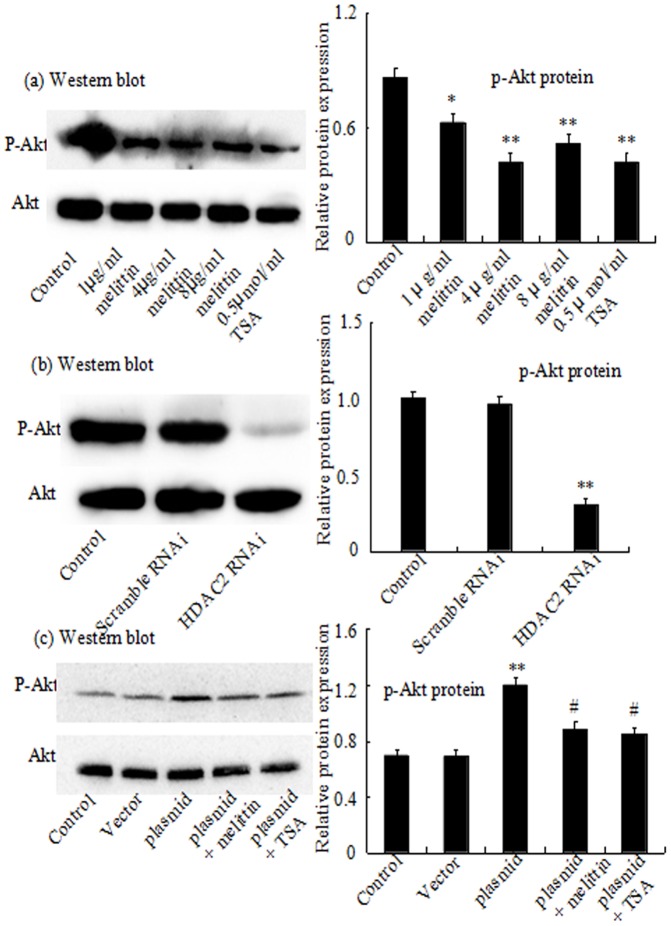
Effects of PTEN on Akt activation in HepG2 cells. HepG2 cells were treated with 1, 4, 8 µg/ml of melittin and 0.5 µmol/ml TSA for 24 h. The p-Akt and Akt proteins expression were analyzed by Western blot (a). *P<0.05, **P<0.01 compared to cells without melittin treatment. Silencing of HDAC2 genes in HepG2 cells was performed and phosphorylation of Akt was checked by Western blot (b) as described in the Materials and methods section. Representative images of at least three independent experiments are shown and results of densitometric analyses (mean±SE) are shown. **P<0.01 vs controls and scrambled siRNA. HepG2 cells were treated with pEGFP-C2, pEGFP-C2-HDAC2 plasmid, pEGFP-C2-HDAC2 plasmid and melittin, pEGFP-C2-HDAC2 plasmid and TSA for 24 h. Phosphorylation of Akt was checked by Western blot (c). **P<0.01 vs control and vector group. ^#^P<0.05 vs pEGFP-C2-HDAC-2 plasmid group.

Akt is a downstream receptor of PI3K that is known to mediate survival signaling. To determine whether melittin-inhibited cell proliferation is closely related to an Akt signal, we examined the phosphorylation levels of Akt in cells following melittin treatment for 24 h. It was demonstrated that (Fig 8 a), melittin did not affect total Akt protein levels; however, Akt phosphorylation decreased in comparison to control after treatment of melittin for 24 h. Knockdown of HDAC2 significantly prevented the activation of Akt comparied to control or scrambled siRNA (Fig 8 b). To further investigate the interaction between HDAC2 and melittin in the regulation of activation of PI3K/Akt signaling, we overexpressed HDAC2 and treated with melittin or TSA in HepG2 cells. The study revealed that the phosphorylation level of Akt was obviously elevated, while the total protein expression level of Akt did not changed. However, the phosphorylation level of Akt was reduced under melittin or TSA in comparison to pEGFP-C2-HDAC2 plasmid, while it was still higher than the control and vector groups(Fig 8 c). These results suggest that there might be a deacetylation occurred in the PTEN gene which contributes to the increase in PI3K/Akt activation and melittin downregulated the expression of phosphorylation Akt, suppressed the activation of Akt.

## Discussion and Conclusions

Mutation or other inactivation of PTEN is a common feature of many types of tumor [18], [19], including those of the brain, breast, prostate, lung, liver and gastric carcinoma [18], [20]–[22]. Low expression of PTEN function increases the cellular concentration of PIP-3, which in turn leads to Akt activation, suggesting that the function of PTEN is exerted through the negative regulation of the PI3K/Akt pathways and consequently leads to cancer cell proliferation and ultimately stimulates tumor genesis [23]. PTEN is tightly controlled by various non-genomic mechanisms [24]. In this study, aberrant PTEN expression can result from epigenetic medications including histone deacetylation [25], [26].

Acetylation of histones and non-histone proteins pivotally modulates gene expression and signaling. Acetylated histones contribute to an epigenetic mechanism marking transcriptionally active regions of chromatin [27]. The HATs and HDACs dynamically regulate many genes associated with cellular proliferation. Histone acetylation by HATs induces gene activation, while deacetylation by HDACs mediates transcriptional repression [28]. High expression of HDAC2 has also been detected in oral, prostate, ovarian, breast or gastric cancer [29]-[31]. Previous studies have shown that HDAC2 overexpression induces cells proliferation in various cancers such as gastric cancer, cervical cancer, breast cancer, colorectal cancer and liver cancer cells [11], [32]. Even more intriguing, recent study suggested that inhibition of HDAC enhanced the expression of PTEN [7], [33]. HDAC inhibitors, such as trichostatin A (TSA), have been demonstrated to inhibit the expression of HDAC and upregulate PTEN [34]. Here, we found that low expression of PTEN was observed in HepG2 cells without treatment, whereas treatment with melittin and TSA triggered a raise on PTEN expression. The results of the HDAC2 expression profile indicate that the expression of HDAC2 are lower than in HepG2 cells treated with melittin and TSA compared with control. Meanwhile, the result also revealed that histone complex H3 acetylation was valuated under melittin and TSA.

Date from study has been demonstrated that PTEN inhibits cell growth and induces cell cycle arrest through the PI3K/Akt pathway [35]. In addition, Moon et.al have been reported that melittin enable to trigger the downregulation of Akt phosphorylation [36]. In this study, the expression of Akt phosphorylation under melittin is consistent with the report. In my current study, we discover melittin could enhance the level of PTEN via the PI3K/Akt pathway, which may be contact with the downregulation of HDAC2. Our findings also support that the HDAC2 expression is negatively correlated with PTEN expression. Knockdown of HDAC2 by siRNA inhibits cell proliferation and restores PTEN gene expression. The PI3K/Akt pathway which is activated by various cytokines and integrin signaling, plays an important role in controlling cancer cell growth and survival [37], [38]. Low expression of HDAC2 causes the upregulation of PTEN, thereby inhibiting Akt phosphorylation and resulting in downregulation of CyclinD1 expression. Ultimately, this process results in the arrest of most HepG2 cells at the G0/G1 phase, negatively regulating cell cycle progression. This observation is consistent with previous reports that melittin can arrest cells at G0/G1 phase. Moreover, we notice that knockdown of HDAC2 gene using siRNA enhances the inhibition of cell proliferation and accelerated PTEN transcription and translation, inhibits Akt phosphorylation, and prevents cell progression from G0/G1 phase into the G2/M phase and S phase.

**Figure 9:**
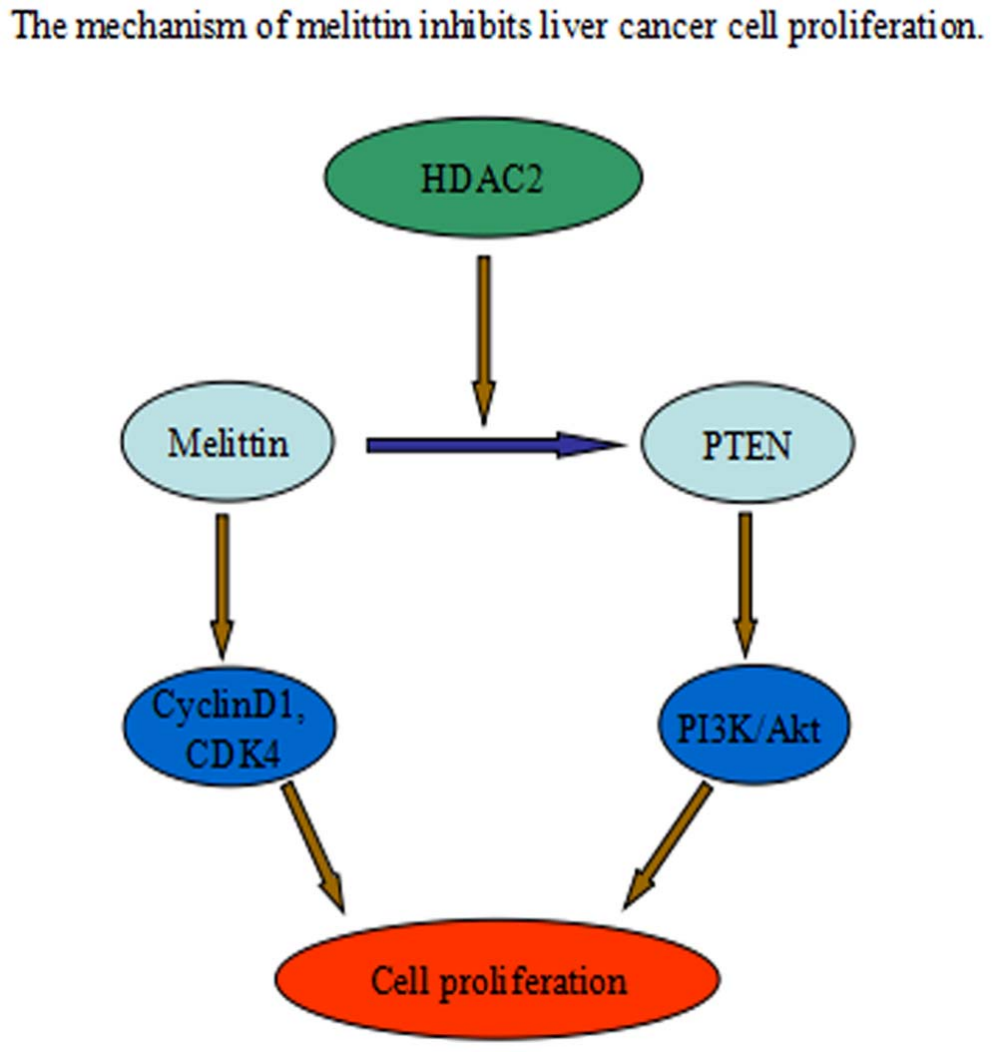
The mechanism of melittin inhibits liver cancer cell proliferation. Melittin downregulates the expression of HDAC2. Then, inhibition of HDAC2 leads to an increased levels of PTEN and promotes Akt inactivation, inhibition of the PI3K/Akt signaling pathways, thereby inhibits cell proliferation.

In summary, melittin may exert anti-proliferation activity. Our experiment results tested the hypothesis that melittin may have inhibitory effects in HepG2 cell cycle progression through HDAC2-mediated PTEN upregulation, Akt inactivation, and inhibition of the PI3K/Akt signaling pathways (Fig 9).
